# Developmental expression of three *prmt* genes in *Xenopus*

**DOI:** 10.24272/j.issn.2095-8137.2018.064

**Published:** 2018-08-20

**Authors:** Cheng-Dong Wang, Xiao-Fang Guo, Thomas Chi Bun Wong, Hui Wang, Xu-Feng Qi, Dong-Qing Cai, Yi Deng, Hui Zhao

**Affiliations:** 1 Key Laboratory for Regenerative Medicine, Ministry of Education, School of Biomedical Sciences, Faculty of Medicine, The Chinese University of Hong Kong, Hong Kong SAR, China; 2 Key Laboratory for Regenerative Medicine of Ministry of Education, Jinan University, Guangzhou Guangdong 510632, China; 3 Guangdong Provincial Key Laboratory of Cell Microenvironment, Department of Biology, South University of Science and Technology of China, Shenzhen Guangdong 518055, China; 4 Kunming Institute of Zoology, Chinese Academy of Sciences-The Chinese University of Hong Kong Joint Laboratory of Bioresources and Molecular Research of Common Diseases, Hong Kong SAR, China; 5 Department of Developmental & Regenerative Biology, College of Life Science and Technology, Jinan University, Guangzhou Guangdong 510632, China

**DEAR EDITOR**,

Protein arginine methyltransferases (PRMTs) are involved in many cellular processes via the arginine methylation of histone or non-histone proteins. We examined the expression patterns of *prmt4*, *prmt7*, and *prmt9* during embryogenesis in *Xenopus* using whole-mount *in situ* hybridization and quantitative reverse transcription polymerase chain reaction (RT-PCR). *Xenopus prmt4* and *prmt7* were expressed in the neural crest, brain, and spinal cord, and also detected in the eye, branchial arches, and heart at the tailbud stage. Specific *prmt9* signals were not detected in *Xenopus* embryos until the late tailbud stage when weak expression was observed in the branchial arches. Quantitative RT-PCR indicated that the expression of *prmt4* and *prmt7* was up-regulated during the neurula stage, whereas *prmt9* maintained its low expression until the late tailbud stage, consistent with the whole-mount *in situ* hybridization results. Thus, the developmental expression patterns of these three *prmt* genes in *Xenopus* embryos provide a basis for further functional study of such genes.

Post-translational modification plays an essential role in modulating the structure and function of a protein (Walsh & Jefferis, 2006). Arginine methylation is a common post-translational modification in vertebrates, and is mediated by protein arginine methyltransferases (PRMTs) (Biggar & Li, 2015; Carr et al., 2015). PRMTs can catalyze the transfer of a methyl group from S-adenosylmethionine (SAM) to the guanidine nitrogen atoms of arginine to form methylarginine (Herrmann et al., 2009). Based on the number and symmetry of the methyl group in methylarginine, PRMTs can be divided into three categories. PRMT4 (type I PRMT) and PRMT9 (type II PRMT) catalyze the formation of asymmetric and symmetric dimethylarginine, respectively (Cook et al., 2006; Yang & Bedford, 2013), whereas PRMT7 (type III PRMT) catalyzes the formation of monomethylarginine (Feng et al., 2013; Feng et al., 2014).

The PRMT-mediated arginine methylation of histone or non-histone proteins is involved in many cellular processes, including transcriptional regulation, signal transduction, and RNA splicing (Biggar & Li, 2015; Carr et al., 2015; Yang et al., 2015). PRMT4, also known as coactivator associated arginine methyltransferase 1 (CARM1), can regulate the cell cycle through arginine methylation of the retinoblastoma protein tumor suppressor (Kim et al., 2015). PRMT9 can methylate SAP145, a component of the U2 snRNP involved in the early stages of splicing, with attenuation of PRMT9 also known to cause gross changes in RNA splicing (Yang et al., 2015).

PRMT7 is required for the maintenance of the regeneration capacity of muscle stem cells by regulating the DNMT3b/p21 axis (Blanc et al., 2016). Specific knockout of *PRMT7* in muscle stem cells can cause elevated expression of CDK inhibitor p21CIP1 and reduced expression of its repressor, DNMT3b, leading to cell-cycle arrest and premature cellular senescence, which can be rescued by restoration of DNMT3b (Blanc et al., 2016). Both *prmt4* and *prmt5* play a combinatorial role during zebrafish myogenesis by controlling fast and slow muscle fiber formation (Batut et al., 2011). Furthermore, *prmt4* is also suggested to regulate the expression of myogenic microRNAs directly (Mallappa et al., 2011).

Although PRMTs are widely involved in various cellular processes via catalyzing the methylation of target proteins, their roles in embryonic development are not yet well understood. Limited studies have shown that PRMT4 and PRMT7 are involved in myogenesis (Batut et al., 2011; Blanc et al., 2016; Mallappa et al., 2011). *Xenopus* is an excellent model in developmental biology (Harland & Grainger, 2011), and all *prmt* genes (*prmt1*–*9*) have been identified in the *Xenopus tropicalis* genome. In this study, we selected three *prmt* genes, that is, *prmt4*, *prmt7,* and *prmt9*, and studied their spatial and temporal expression patterns during the embryonic development of *Xenopus*. Our study will provide a basis for further investigations on the functions of *prmt* genes in *Xenopus*.

We searched *prmt* genes of *Xenopus tropicalis* and other species in the NCBI database (Supplementary Table S1, available online). Protein sequence alignments were performed using Geneious v4.8.5 (www.geneious.com/previous-versions/#geneious-4-dot-8), with a dendrogram tree then constructed using neighbor-joining in the same program.

*Xenopus laevis prmt4* (NM_001094676), *prmt7* (NM_001086541), and *prmt9* (NM_001096961) sequences were obtained by searching the NCBI database. The open reading frames (ORFs) of *prmt4*, *prmt7*, and *prmt9* were amplified using reverse transcription polymerase chain reaction (RT-PCR). The PCR products were subcloned into the pBluescript II KS (+) vector and verified by sequencing. To prepare probes for *in situ* hybridizations, plasmids were linearized by cutting with *Xho*1 and used as templates for the synthesis of digoxigenin-labeled anti-sense probes with T7 RNA polymerase (Roche, Indianapolis, USA).

*Xenopus laevis* embryos were collected, cultured, and fixed as described previously (Wang et al., 2011). Whole-mount *in situ* hybridization was performed according to standard methods (Harland, 1991). After *in situ* hybridization, the embryos were embedded and sectioned at a thickness of 50 μm. Detailed information on the vibratome sections is described in our previous study (Kam et al., 2010).

Total RNA was extracted from *Xenopus tropicalis* embryos using TRIzol reagent (Molecular Research Center Inc., USA). The cDNA was synthesized using the ReverTra Ace^®^ qPCR RT Kit (Toyobo, Japan). Quantitative PCR was performed using the SYBR^®^ Green real-time PCR master mix (Toyobo, Japan). The primer sequences used are listed in Table 1. *Ornithine decarboxylase* (*odc*) was used as the internal control.

**Table 1:** Primers for quantitative RT-PCR

Gene	Sequence (5′–3′)
*prmt1*	Fw: CAAACATGGCTGAAGCGAGC
	Re: ACATCTCCTCATGGATGCCAAAG
*prmt2*	Fw: ACAGCCTGCATTCACTTGGT
	Re: TAATCAGGCTTGGGTCTGGC
*prmt3*	Fw: AAGATGTGGATCTGCCCGTG
	Re: CAGGTATCGGGGTACACTGC
*prmt4*	Fw: GGAGATCCAGAGACACGCTG
	Re: TGCATTTGAACACGCAGACG
*prmt6*	Fw: GGCCAGTAGTATGTCCCAGC
	Re: GCGTACCCCATCCATTCACT
*prmt7*	Fw: TGCGTGTGGTACAGCCTAAC
	Re: AATGACATGCAGGACGCTCT
*prmt9*	Fw: TTGATGCAGGCTTGTTTGGC
	Re: TCTGGGCACTCTACTGCCAT
*odc*	Fw: GGGCAAAAGAGCTTAATGTGG
	Re: TGCCAACATGGAAACTTACA

**Figure 1:**
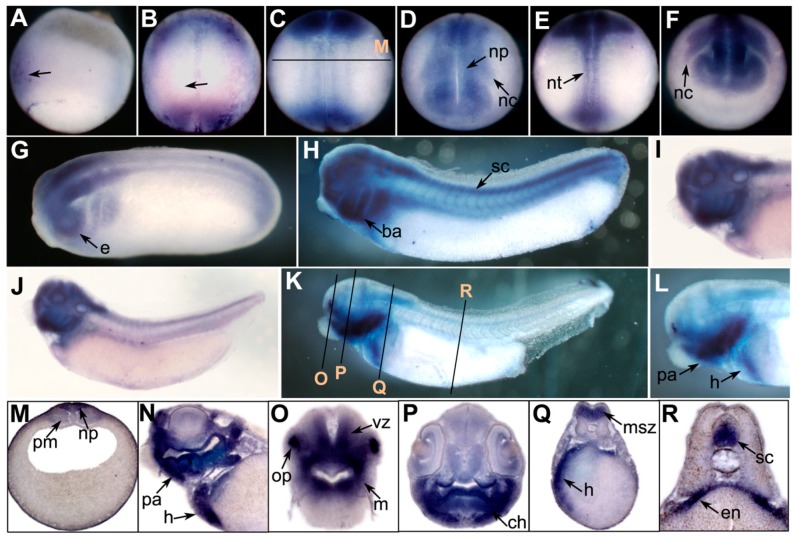
Spatial expression of *prmt4* in *Xenopus* embryos

We performed whole-mount *in situ* hybridization to examine the spatial expression of *prmt* genes in *Xenopus* embryos. No evident signals were detected before gastrulation. In the early gastrula stage, the *prmt4* signal was mainly expressed in the dorsal ectoderm, which gives rise to the neural ectoderm (Figure 1A). At the early neurula stage, the *prmt4* signal was enriched in the anterior region of the neural plate as well as the posterior region around the blastopore (Figure 1B). After this, *prmt4* was strongly expressed in the neural plate and neural crest (Figure 1C, D). This expression pattern persisted when neural crest migration began in the later neurula stage (Figure 1E, F). During the tailbud stage, *prmt4* was detected throughout the central nervous system, including the brain and spinal cord (Figure 1G, H). Strong expression was also observed in the branchial arches and eye vesicles (Figure 1G, H). In stage 35, *prmt4* expression was enriched in the head region, including the forebrain, midbrain, hindbrain, eye, and branchial arches (Figure 1I, J). In stage 40, *prmt4* was expressed in the olfactory placode, jaw, and heart, and weak expression in the paraxial mesoderm was also observed (Figure 1K–R). Weak signals were also detected in the eye, brain, dorsal region of the endoderm, and spinal cord (Figure 1N–R).

A: Lateral view of a stage 10 embryo with dorsal toward the left showing differential expression of *prmt4* along the dorsoventral axis. Arrow indicates signal on the dorsal side. B–F: *prmt4* signal detected in the neural crest and neural plate during the neurula stage. B: Stage 15 embryo; C, D: Stage 18 embryos; E, F: Stage 20 embryos. B, C, E: Dorsal view with anterior toward the top. D, F: Anterior view with dorsal toward the top. G–L: Expression of *prmt4* detected in the brain, spinal cord, branchial arches, and heart anlage. G, H, J, K: Lateral view of stage 25 (G), stage 28 (H), stage 35 (J), and stage 40 (K) embryos. Enlarged head regions of embryos in J and K are shown in I and L, respectively. M: Transverse section of embryo at the level illustrated by the black line in C. N: Longitudinal section of a stage 40 embryo. O–R: Transverse sections of embryo at the levels illustrated by the black lines in K. ba: branchial arches; e: eye; en: endoderm; ch: second pharyngeal arch; h: heart; m: first pharyngeal (maxillary) arch; msz: marginal and subventricular zones of midbrain and neural tube; nc: neural crest; np: neural plate; nt: neural tube; op: olfactory placode; pa: pharyngeal arch; pm: paraxial mesoderm; sc: spinal cord; vz: ventricular zone of neural tube.

**Figure 2:**
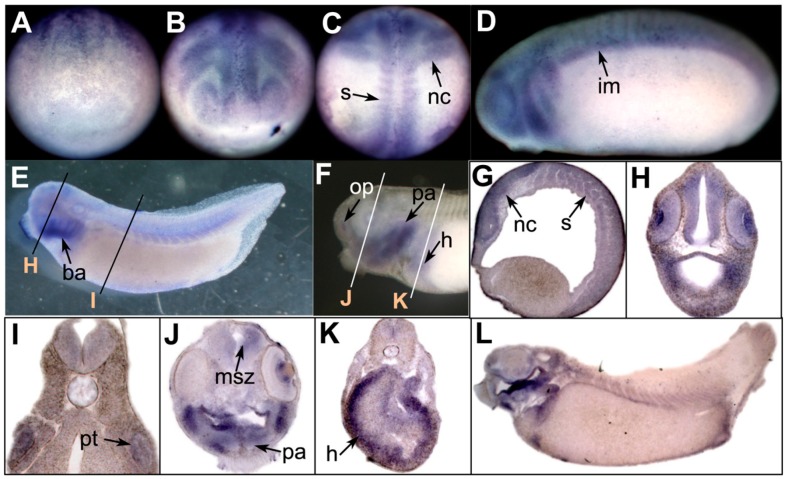
Expression of *prmt7* in *Xenopus* embryos

No apparent expression of *prmt7* was detected in the embryos at the early gastrula stage (data not shown). In the early neurula stage, *prmt7* was weakly expressed in the neural plate (Figure 2A). The expression of *prmt7* intensified and expanded to the migrating neural crest, anterior neural tube, and somites (Figure 2B, C), and then showed strong expression in the branchial arches during the tailbud stage (Figure 2D, E). At stage 25, *prmt7* expression was also detected in the intermediate mesoderm (Figure 2D). At stage 29, *prmt7* signals were detected in the brain, eye, and somites (Figure 2E–H). Weak expression was also observed in the pronephric tubule (Figure 2I). At stage 40, *prmt7* was strongly expressed in the branchial arches, with weak signals in the heart, eye, brain, and olfactory placode (Figure 2J–L).

A: Weak signal of *prmt7* detected on the neural fold of stage 15 embryo. Anterior view with dorsal toward the top. B, C: *prmt7* expressed in migrating neural crest, neural tube, and somites in a stage 20 embryo. B: Anterior view with dorsal toward the top; C: Dorsal view. D, E: Expression of *prmt7* in branchial arches, intermediate mesoderm, brain, and eye in a stage 25 embryo (D). At later stage (stage 29, E), strong *prmt7* signals remained at branchial arches. Faint *prmt7* expression detected in pronephric tubule and in somites. F: *prmt7* signals detected in the eye, pharyngeal arches, and heart at stage 40. G: Longitudinal section of a stage 20 embryo showing *prmt7* signals the in neural crest and somites. H, I: Transverse sections of a stage 29 embryo at the levels illustrated by the black lines in E. J, K: Transverse sections from a stage 40 embryo at the levels illustrated by the white lines shown in F. L: Longitudinal section of a stage 40 embryo. ba: branchial arches; h: heart; im: intermediate mesoderm; msz: marginal and subventricular zones of midbrain and neural tube; nc: neural crest; op: olfactory placode; pa: pharyngeal arch; pt: pronephric tubule; s: somite.

Whole-mount *in situ* hybridization could not detect specific signals of *prmt9* in *Xenopus* embryos at almost every stage, though a weak signal was observed in the branchial arches at the late tailbud stage (data not shown).

**Figure 3:**
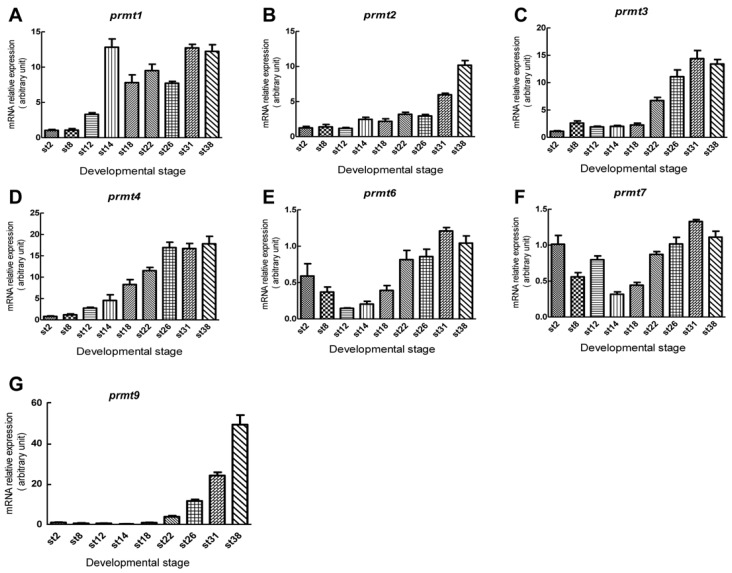
Quantitative RT-PCR analysis of *prmt* gene expression in *Xenopus tropicalis* embryos

We studied the temporal expression patterns of *Xenopus prmt* genes using quantitative RT-PCR. Different from pseudotetraploid *Xenopus laevis*, the diploid *Xenopus tropicalis* species has two sets of chromosomes. Thus, we collected *Xenopus tropicalis* embryos at different stages and measured the mRNA expression levels of *prmt1–4*, *prmt6*, *prmt7*, and *prmt9* (Figure 3). Maternal expression of *prmt6* and *prmt7* was detected, which decreased during the cleavage stage (Figure 3E, F). Apart from *prmt7,* the expression levels of the detected *prmt* genes were low during the gastrula stage (Figure 3F). The expression of *prmt4*, *prmt6*, and *prmt7* were gradually up-regulated from the neurula stage (Figure 3D–F). However, the expression levels of *prmt2*, *prmt3*, and *prmt9* increased from the tailbud stage (Figure 3B, C, G). During the late tailbud stage, most of the *prmt* genes maintained their high expression levels, except for *prmt2* and *prmt9*, whose expression continued to increase (Figure 3B, G). The expression of *prmt9* remained low before the late tailbud stage (Figure 3G). In contrast, *prmt1* expression reached a high level at the early neurula stage (Figure 3A). The up-regulation in the expression of *prmt4* and *prmt7* during neurulation (Figure 3D, F) accords with their enhanced staining of embryos, as revealed by whole-mount *in situ* hybridization (Figure 1B–F; Figure 2A*–*C). The low expression level of *prmt9* before the late tailbud stage (Figure 3G) is consistent with the slight staining of embryos after *in situ* hybridization.

**Figure 4:**
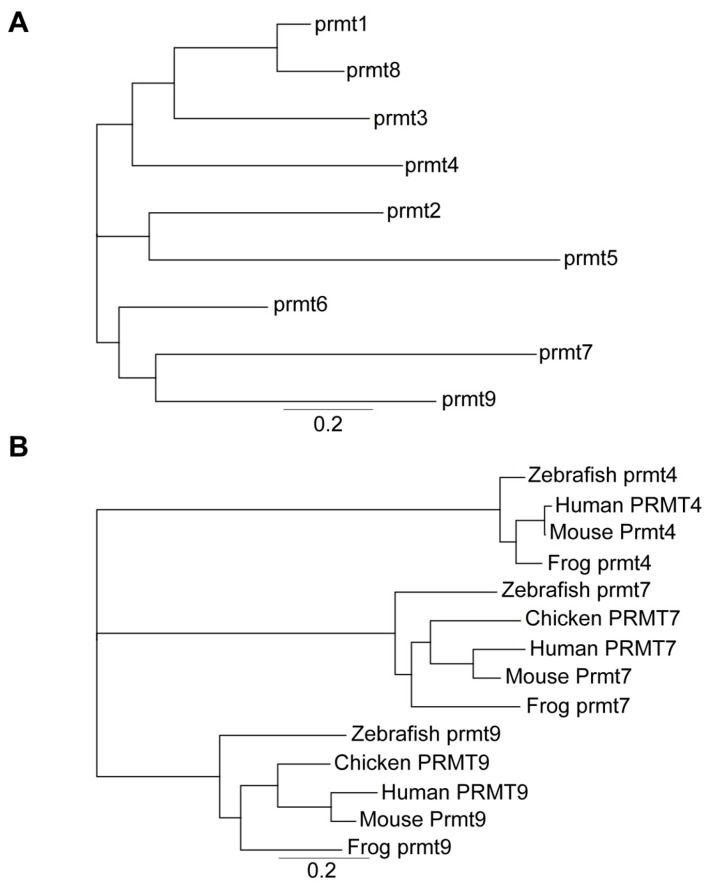
Phylogenetic analysis of prmt proteins

Searching the NCBI database we found that all *prmt* genes, including *prmt1–9*, have been identified in *Xenopus tropicalis*. A phylogenetic tree based on their protein sequence alignments was generated (Figure 4A). Results showed that type I PRMTs (prmt1, prmt2, prmt3, prmt4, prmt6, and prmt8) exhibited fewer genetic changes, whereas prmt5 and prmt7 demonstrated more significant genetic variation (Figure 4A). We conducted phylogenetic analysis of the *Xenopus* prmt4, prmt7, and prmt9 proteins to illustrate their evolutionary distances to humans, mice, chickens, frogs, and zebrafish (Figure 4B).

mRNA expression levels of *Xenopus prmt1–4* (A*–*D), *prmt6* (E), *prmt7* (F), and *prmt9* (G) were measured in different embryonic stages, including cleavage (stage 2), blastula (stage 8), gastrula (stage 12), neurula (stages 14, 18), early tailbud (stages 22, 26), and late tailbud (stages 31, 38), *ornithine decarboxylase* (*odc*) was used as an internal control.

A: prmt1–9 proteins of *Xenopus tropicalis*. B: prmt4, prmt7, and prmt9 proteins from humans (*Homo sapiens*), mice (*Mus musculus*), chickens (*Gallus gallus*), frogs (*Xenopus tropicalis*), and zebrafish *(Danio rerio*).

We examined the expression patterns of three *prmt* genes during embryonic development. Our results indicated that *prmt4* and *prmt7* showed similar expression patterns. Both were expressed in the neural plate during neurulation and were then detected in the brain and spinal cord (Figure 1 and Figure 2). These data suggest that *prmt4* and *prmt7* function in neural development, similar to that found in other PRMT members (Batut et al., 2005; Hashimoto et al., 2016; Honda et al., 2017; Lee et al., 2005; Lee et al., 2017; Lin et al., 2013). Furthermore, *prmt4* and *prmt7* were both expressed in the neural crest, which has not been reported previously. Apart from the brain, *prmt4* and *prmt7* were also expressed in other regions of the head, including the eye and branchial arches. At the late tailbud stage, both were detected in the olfactory placode, pharyngeal arches, and heart regions (Figure 1K, N–R; Figure 2F, J–L). In zebrafish, the expression of *prmt8* has also been detected in the heart at the later stages (Lin et al., 2013). Here, weak *prmt4* signals were detected in the paraxial mesoderm (Figure 1M), whereas *prmt7* was evidently expressed in the somites (Figure 2C, G). These results are in agreement with their potential roles in myogenesis, which have been studied to some extent in zebrafish (Batut et al., 2011). In addition, *prmt4* and *prmt7* also shared similar temporal expression patterns at the neurula and tailbud stages when their expression levels were gradually up-regulated (Figure 3D, F). The high similarity in expression patterns between *prmt4* and *prmt7* implies that different members of the PRMT family may have redundant roles in regulating early embryonic development. Compared with other examined *prmt* genes, *prmt9* exhibited a distinct temporal expression pattern. Its expression level was very low before the early tailbud stage but was dramatically elevated from the late tailbud stage (Figure 3G). This is in line with the *in situ* hybridization results, in which specific *prmt9* signals were not detected until the late tailbud stage. Although prmt9 is a non-histone methyltransferase involved in regulating RNA splicing (Yang et al., 2015), whether the special expression pattern of *prmt9* is related to its role in RNA splicing remains to be illustrated. This study will facilitate further functional study of *prmt* genes during embryonic development.
